# Safety profiles of doxycycline, minocycline, and tigecycline in pediatric patients: a real-world pharmacovigilance analysis based on the FAERS database

**DOI:** 10.3389/fphar.2024.1413944

**Published:** 2024-07-26

**Authors:** Yanli Qiao, Yechao Chen, Qiaoyun Wang, Jingrui Liu, Xiaohui Guo, Qiaoling Gu, Peng Ding, Haixia Zhang, Hongliang Mei

**Affiliations:** ^1^ Department of Pharmacy, Nanjing Drum Tower Hospital, School of Basic Medicine and Clinical Pharmacy, China Pharmaceutical University, Nanjing, Jiangsu, China; ^2^ School of Basic Medicine and Clinical Pharmacy, China Pharmaceutical University, Nanjing, Jiangsu, China; ^3^ School of Pharmacy, Faculty of Medicine, Macau University of Science and Technology, Taipa, Macau SAR, China; ^4^ Department of Pharmacy, Nanjing Drum Tower Hospital, Affiliated Hospital of Medical School, Nanjing University, Nanjing, Jiangsu, China

**Keywords:** tetracyclines, children, pharmacovigilance, FAERS, reporting odds ratio

## Abstract

**Introduction:**

Recently, the rise of antibiotic resistance has prompted a reconsideration of tetracyclines. However, existing studies are inadequate in assessing the pediatric safety of this class of antibiotics. To address the gap, our study aims to comprehensively assess the safety of tetracyclines in children.

**Methods:**

Adverse event (AE) reports from January 2005 to September 2023 were obtained from the U.S. Food and Drug Administration (FDA) Adverse Event Reporting System (FAERS) database, and reporting odds ratio (ROR) was performed to identify potential risk signals in children under 18 years old who were administered any of the three tetracyclines: doxycycline, minocycline, and tigecycline.

**Results:**

A total of 1903 AE cases were included in our study: 782 for doxycycline, 981 for minocycline, and 140 for tigecycline. Doxycycline and tigecycline were predominantly associated with “general disorders and administration site conditions” and “gastrointestinal disorders,” while minocycline was more frequently linked to “skin and subcutaneous tissue disorders” and “gastrointestinal disorders.” Psychiatric risks predominantly included depression, suicidal ideation, and suicide attempt. In the category of skin and subcutaneous tissues, 30.88% of the minocycline-induced drug reaction with eosinophilia and systemic symptoms (DRESS) cases resulted in death, alongside a high occurrence of co-occurring AEs such as multiple organ dysfunction syndrome, Type 1 Diabetes Mellitus (T1DM), and autoimmune thyroiditis. As for the endocrine system, both doxycycline and minocycline were found to potentially increase the risk of thyroid dysfunction. For children under the age of 8, doxycycline was associated with tooth discoloration (N = 7, ROR = 20.11%, 95% CI: 9.48–42.67), although it remained unclear whether the discoloration was permanent.

**Conclusion:**

Our findings indicated that for pediatric patients, the majority of results were in line with the prescribing information and previous studies, and minocycline tended to cause more frequent and severe AEs than doxycycline. However, it is noteworthy that exceptions were found for psychiatric disorders and thyroid dysfunction associated with doxycycline, which are not mentioned in its FDA prescribing information. Additionally, further safety studies on tigecycline are still needed for children. When prescribing tetracyclines to pediatric patients, a careful risk-benefit assessment is crucial.

## 1 Introduction

Tetracyclines are broad-spectrum antibiotics, exhibiting activity against a wide range of Gram-positive and Gram-negative bacteria, atypical organisms such as *Chlamydia*, *Mycoplasma*, Spirochetes, and *Rickettsia*, as well as protozoan parasites, and the mechanism of action involves inhibiting protein synthesis by preventing the attachment of aminoacyl-tRNAs to the A site of the 30S subunit of ribosomes (Chopra and Roberts, 2001). In addition to their antibacterial activity, tetracyclines can also inhibit metalloproteinases and exhibit anti-inflammatory, antiapoptotic, and antioxidant effects (Orylska-Ratynska et al., 2022).

The first tetracycline antibiotic, chlortetracycline, was first reported in the scientific literature in 1948 (Bryer et al., 1948). Following the initial breakthrough, several tetracyclines, including tetracycline, doxycycline, and minocycline, became staples in antimicrobial therapy. Despite their initial success, the rise of tetracycline-resistant bacteria and the introduction of alternative antibiotics, such as cephalosporins and fluoroquinolones, led to a decline in the use of tetracyclines (Chopra and Roberts, 2001; Emmerson and Jones, 2003; LaPlante et al., 2022). The clinical landscape remained stagnant for over 3 decades following the U.S. Food and Drug Administration (FDA) approval of minocycline in 1971, with no new tetracyclines entering the market.

With the rise of antibiotic multi-drug resistance, the imperative to combat this global health challenge has reignited interest in the tetracycline class. The FDA approval of third-generation tetracyclines—tigecycline in 2005, eravacycline and omadacycline in 2018—marks a promising resurgence (Wenzel et al., 2005; Zhanel et al., 2016; Watkins and Deresinski, 2019). Moreover, tetracyclines remain highly effective against atypical pathogens, necessitating a reevaluation of their role in current medical practices (Smilack, 1999).

However, there is a notable scarcity of comprehensive safety studies on tetracyclines in pediatric patients. Currently, doxycycline and minocycline are predominantly prescribed for treating moderate to severe inflammatory acne in children (Zaenglein et al., 2016). However, due to limited research and concerns about tooth discoloration, their use is contraindicated in children under 8 years old (Patheon Pharmaceuticals Inc, 2017; Mayne Pharma, 2022). Tigecycline is not advised for children due to the potential for higher all-cause mortality rates observed in adult trials (McGovern et al., 2013; Shen et al., 2015; Pfizer Inc, 2020). Additionally, the safety and efficacy of omadacycline and eravacycline in pediatric patients have not yet been established (Paratek Pharmaceuticals, 2021; Tetraphase Pharmaceuticals, 2018). Nonetheless, because of the scarcity of other effective antimicrobials, tigecycline has become a useful alternative as a drug of last resort for serious infections, including in children, caused by multi-drug resistant (MDR) and extensively drug-resistant organisms (XDR) (Song et al., 2018). For children under 8 years old, doxycycline and minocycline may be necessary when treating infections like macrolide-resistant *Mycoplasma pneumoniae* (Chou et al., 2019; Ishiwada et al., 2023; National Health Commission of the People’s Republic of China, 2023). Additionally, doxycycline is considered the first-line treatment for Lyme disease and Rocky Mountain spotted fever in children of all ages (American Academy of Pediatrics, 2018; Lantos et al., 2021; Meissner and Steere, 2022). To fill the gap in safety knowledge for pediatric use of tetracyclines, our study focused on the safety profiles of doxycycline, minocycline, and tigecycline, which are more commonly prescribed to this age group. We utilized data from the FDA’s Adverse Event Reporting System (FAERS) for this analysis.

## 2 Methods

Data Source: FAERS is one of the world’s largest spontaneous reporting databases for adverse reactions to marketed drugs and therapeutic biologics. It contains a vast number of adverse event (AE) reports utilized for post-marketing safety monitoring, excelling in detecting serious and rare adverse drug event signals (Rodriguez et al., 2001). The majority of reports come from healthcare professionals, patients, and pharmaceutical manufacturers. The coding of these reports employs the MedDRA (Medical Dictionary for Regulatory Activities) (Version 26.1) preferred terms (PTs). In MedDRA, while a single PT can be associated with multiple system organ classes (SOCs), it is linked to only one primary SOC. In our study, the primary SOC was utilized to categorize PTs.

Study Design: This study collected data from the FAERS database covering the period from January 2005 to September 2023. To eliminate duplicates, the study followed the FDA guidelines: if CASEIDs (identifiers for FAERS cases) were the same, the report with the latest FDA_DT (the date the FDA received the case) was retained, and if both the CASEID and FDA_DT were the same, the report with the higher PRIMARYID (the unique identifier for FAERS reports) was selected (Wei et al., 2022).

The study focused on three tetracyclines: doxycycline, minocycline, and tigecycline, targeting pediatric patients under 18 years old. Only AE reports with reported roles of drugs as “suspect” (either the primary or the secondary suspect) were included. Additionally, a drug event combination was established by combining all AE reports associated with each of the three drugs. Given the lack of uniform standards for drug naming within the FAERS system, coupled with the diverse backgrounds of reporters and the variety of report formats, multiple names for the same drug can exist within the system. Therefore, this study utilized the PharnexCloud database (https://www.pharnexcloud.com/) to search and compile the generic names, brand names, and research codes of these three tetracyclines approved by the FDA as of 30 September 2023. By employing the compiled list (Supplementary Table S1) to conduct searches in the FAERS database, the objective was to extract relevant drug data as comprehensively as possible, thereby ensuring data integrity.

Signal Detection: Based on the disproportionality analysis method, a widely used signal detection method in the pharmacovigilance study, risk signals for the target drugs were mined using the Reporting Odds Ratio (ROR), with the statistical measures being the ROR and its 95% confidence intervals (CI) (Candore et al., 2015; Zhou et al., 2023). The formulas for calculating the ROR and the 95% CI are as follows:

$$ROR=\frac{a/c}{b/d}$$

$${ROR}_{95\%\;CI}=e^{\ln\left(ROR\right)\pm 1.96\sqrt{\frac{1}{a}+\frac{1}{b}+\frac{1}{c}+\frac{1}{d}}}$$

In the formulas, (a) is the count of AE reports of interest related to the suspected drug, (b) is the count of other AE reports for the same suspected drug, (c) is the count of AE reports of interest for all other drugs, and (d) is the count of other AE reports for all other drugs. It was considered statistically significant and deemed a potential signal if a potential risk signal met the following criteria: 1) number of reports≥3, 2) the lower limit of the 95% CI>1. A higher ROR value indicates a stronger association between tetracyclines and AEs.

Statistical Analysis: Descriptive statistics were employed for data analysis. Continuous variables were presented as medians with interquartile ranges (IQR), and categorical variables were reported as frequencies and percentages. Data management and statistical analysis were performed using Microsoft Excel 2021 (Microsoft Corporation, Redmond, Washington, United States) and R software version 4.3.1.

## 3 Results

### 3.1 The baseline characteristics of the children treated with tetracyclines

**FIGURE 1:**
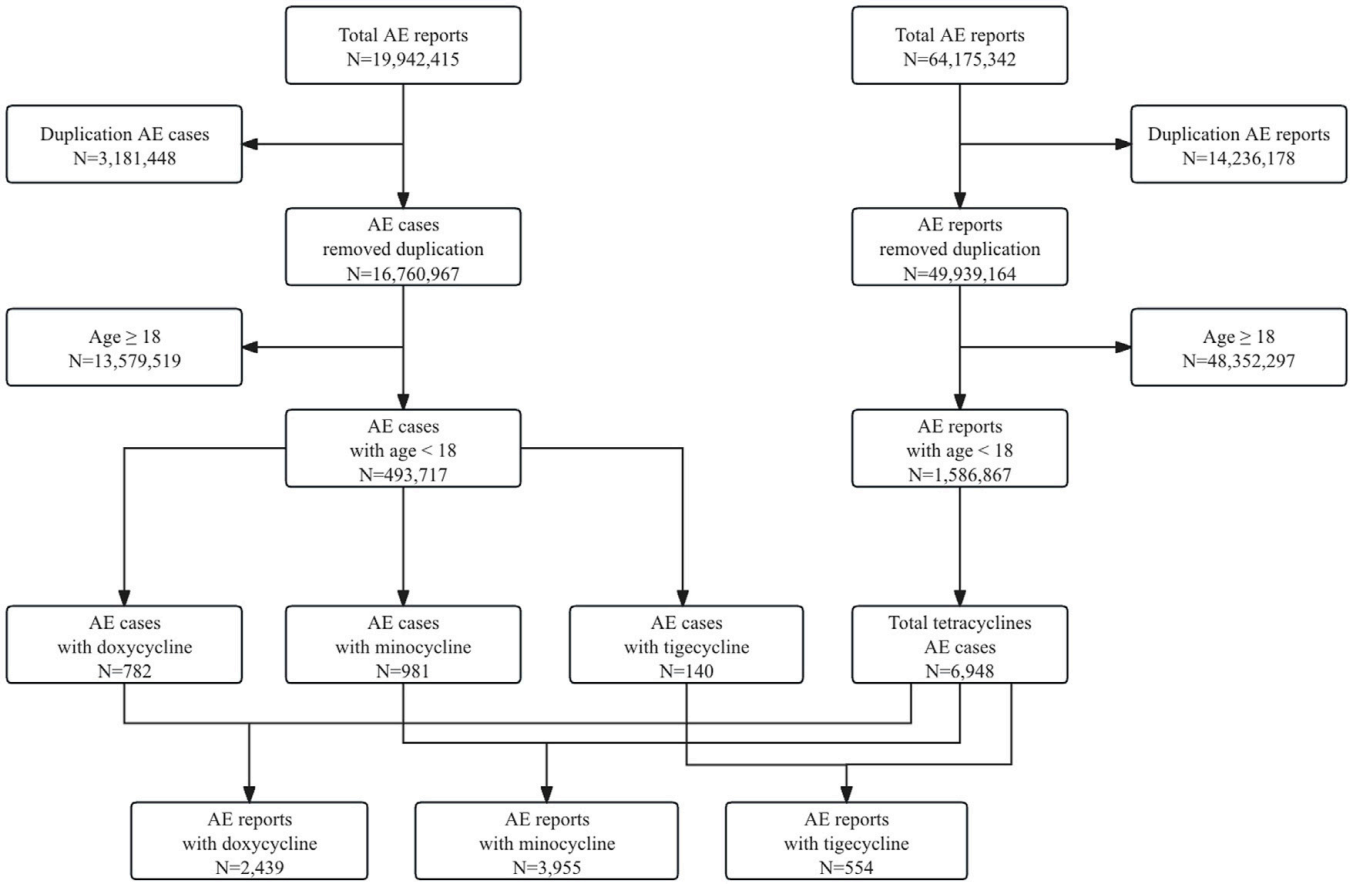
Flowchart of data processing in our study. A detailed description of the selection process of adverse events for doxycycline, minocycline, and tigecycline in pediatric patients in the Food and Drug Administration Adverse Event Reporting System (FAERS).

Between January 2005 and September 2023, FAERS received a total of 19,942,415 AE reports. After deduplication, 16,760,967 reports remained. Out of these, 493,717 pertained to pediatric patients, and ultimately, 6,948 met our inclusion criteria, involving 1,903 cases. Among the drugs studied, minocycline had the highest number of reports (N = 2,439), followed by doxycycline (N = 3,955), and tigecycline (N = 554), with corresponding case counts of 981, 782, and 140, respectively. The detailed data processing workflow is shown in Figure 1.

The baseline characteristics of children treated with doxycycline, minocycline, and tigecycline in FAERS are presented in Table 1. The median age was 7 years for tigecycline (IQR: 5–14 years), 15 years for doxycycline (IQR: 12–16 years), and 16 years for minocycline (IQR: 14–17 years). In terms of gender distribution, the proportion of female patients was slightly higher than male patients, at 52.30% and 57.80%, respectively, for doxycycline and minocycline, whereas tigecycline showed a higher proportion of male patients at 54.29%. The majority of the reports for doxycycline and minocycline were from the United States, accounting for 52.56% and 71.25% respectively, while for tigecycline, China had the highest reports at 19.29%. It is important to note that within FAERS, a single PRIMARYID may correspond to multiple outcomes. For the purpose of baseline statistical analysis, the most severe outcome associated with each PRIMARYID was selected. Notably, over 40% of the reports for all these drugs indicated serious outcomes, including death, life-threatening events, disability, congenital anomaly, or hospitalization, with tigecycline showing the highest incidence at 57.14%. As for pediatric fatalities in FAERS, tigecycline accounted for 23.57%, significantly exceeding those for doxycycline at 2.69% and minocycline at 7.95%. In addition, from 2005 to 2022, we observed a predominant trend of a gradual increase in the number of AE reports per 3-year period.

**TABLE 1:** The baseline characteristics of children treated with doxycycline, minocycline, and tigecycline in FAERS.

Characteristics	doxycycline (N = 782)	minocycline (N = 981)	tigecycline (N = 140)
Age group, (y)			
Median (Q1, Q3)	15 (12,16)	16 (14,17)	7 (5,14)
0–7	103 (13.17%)	15 (1.53%)	71 (50.71%)
8–18	679 (86.83%)	966 (98.47%)	69 (49.29%)
Gender			
Male	362 (46.29%)	399 (40.67%)	76 (54.29%)
Female	409 (52.30%)	567 (57.80%)	62 (44.29%)
Missing	11 (1.41%)	15 (1.53%)	2 (1.43%)
Country			
United States	411 (52.56%)	699 (71.25%)	24 (17.14%)
United Kingdom	82 (10.49%)	41 (4.18%)	9 (6.43%)
Canada	55 (7.03%)	51 (5.20%)	7 (5.00%)
China	1 (0.13%)	2 (0.20%)	27 (19.29%)
Netherlands	2 (0.26%)	0	23 (16.43%)
Outcomes			
Death	21 (2.69%)	78 (7.95%)	33 (23.57%)
Life Threatening	57 (7.29%)	60 (6.12%)	15 (10.71%)
Hospitalization	237 (30.31%)	290 (29.56%)	32 (22.86%)
Disability	14 (1.79%)	15 (1.53%)	0
Required Intervention	2 (0.26%)	12 (1.22%)	0
Other serious events	308 (39.39%)	231 (23.55%)	25 (17.86%)
Unknown	143 (18.29%)	15 (1.53%)	2 (1.43%)
Received year			
2005–2007	39	60	2
2008–2010	45	117	8
2011–2013	52	123	6
2014–2016	152	174	12
2017–2019	177	257	42
2020–2022	250	206	58
2023 (Q1-Q3)	67	44	12
Indication (top5)			
1	Acne(N = 281)	Acne(N = 691)	*Mycobacterium abscessus* Infection (N = 38)
2	Lyme Disease (N = 38)	Confluent and Reticulate Papillomatosis (N = 12)	Mastoiditis (N = 37)
3	Acne Conglobata (N = 26)	Folliculitis (N = 11)	Sepsis (N = 10)
4	Relapsing Fever (N = 15)	Infective Pulmonary Exacerbation of Cystic Fibrosis (N = 8)	Pneumonia (N = 9)
5	Sclerotherapy (N = 13)	*Mycobacterium abscessus* Infection (N = 7)	*Acinetobacter* Infection (N = 6)

(FAERS: the FDA Adverse Event Reporting System).

Further analysis of tigecycline-related fatal cases (N = 33) revealed that 27 cases (81.81%) involved PTs such as drug ineffective, drug ineffective for unapproved indication, treatment failure, drug resistance, intentional product use issue, multiple drug resistance, and product use issue. The median age was 6 years (IQR: 0–8 years). The top three indications were sepsis (N = 8), pneumonia (N = 5), and *Acinetobacter* infection (N = 4). The top three countries with the highest number of fatalities were China (N = 13), Greece (N = 7), and the United Kingdom (N = 4).

Regarding the indications of the AE reports, doxycycline was primarily prescribed for acne (N = 281), Lyme disease (N = 38), and acne conglobata (N = 26); minocycline was most frequently used for acne (N = 691), confluent and reticulated papillomatosis (N = 12), and folliculitis (N = 11); tigecycline was mainly used to treat *Mycobacterium abscessus* infection (N = 38), mastoiditis (N = 37), and sepsis (N = 10).

### 3.2 Tetracycline-related SOCs in pediatric patients

**FIGURE 2:**
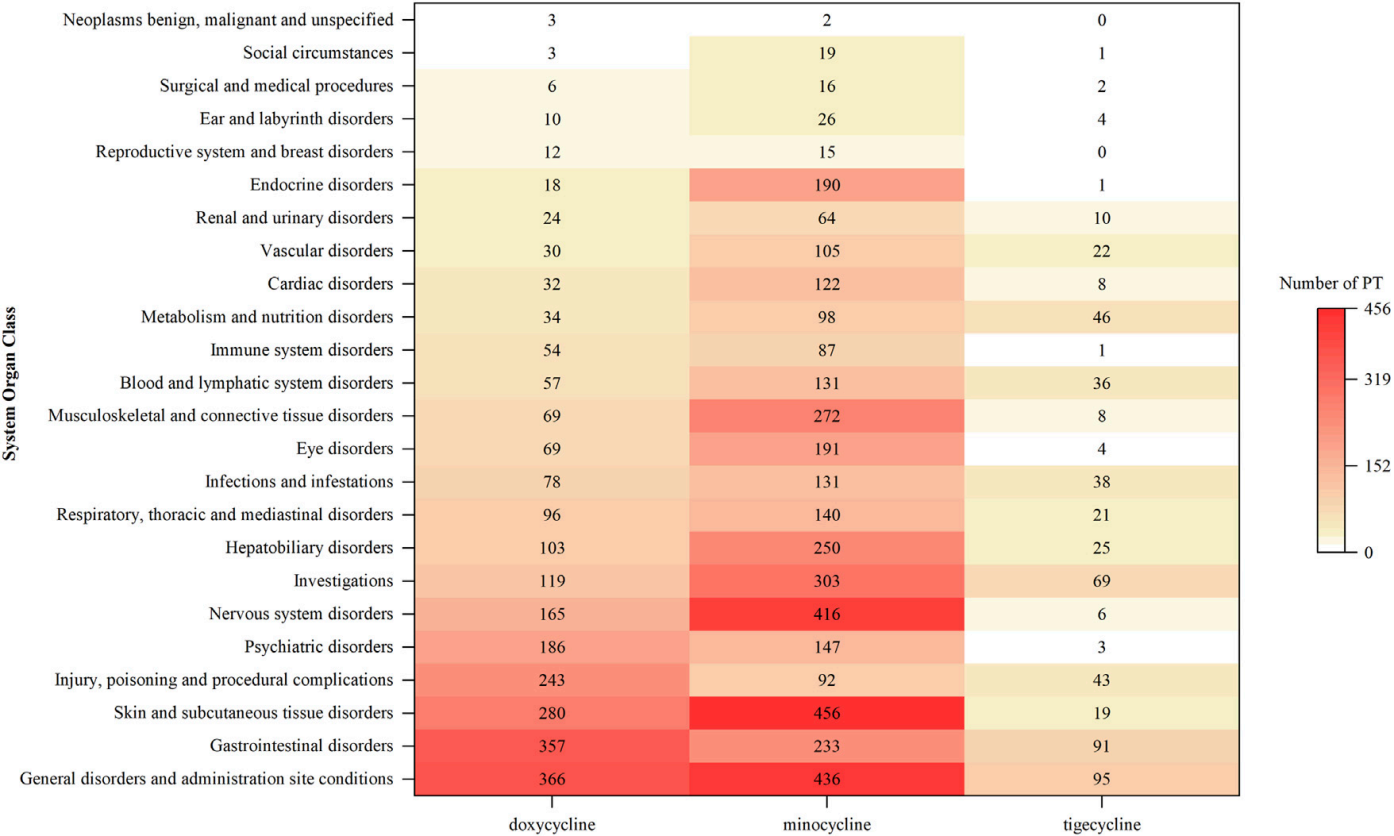
The number of AE reports at SOC levels with significant signal PTs in at least one drug. (AE: adverse event; SOC: system organ class; PT: preferred term).

The number of AE reports at SOC levels with significant signal PTs in at least one drug is detailed in Figure 2. There were 24 SOCs involved in children, excluding “Pregnancy, Puerperium and Perinatal Conditions,” “Product Issues,” and “Various Congenital, Familial, and Genetic Disorders.” The affected SOCs varied slightly among the tetracyclines. Specifically, doxycycline mainly influenced “General Disorders and Administration Site Conditions” (N = 366), “Gastrointestinal Disorders” (N = 357), and “Skin and Subcutaneous Tissue Disorders” (N = 280). Minocycline chiefly impacted “Skin and Subcutaneous Tissue Disorders” (N = 456), “General Disorders and Administration Site Conditions” (N = 436), and “Nervous System Disorders” (N = 416). Tigecycline primarily focused on “General Disorders and Administration Site Conditions” (N = 95), “Gastrointestinal Disorders” (N = 91), and “Various Investigations” (N = 69).

For each drug, we selected the top 20 PTs based on the number of reports that exhibited a significant risk signal and categorized them by SOC. After compiling the results for the three drugs, we listed the ROR for the corresponding PTs of each drug to facilitate comparison, with bold text indicating that the PT is among the top 20 for the drug. The results are presented in Table 2. In Table 2, we excluded signals that were clearly irrelevant to the drugs, such as death, drug ineffective, condition aggravated, treatment failure, drug resistance, sepsis, and off-label use. Additionally, for a comprehensive overview of the significant risk signals associated with each drug, please refer to Supplementary Table S2.

**TABLE 2:** The top 20 PTs (highlighted in bold) with significant risk signals based on the number of AE reports for doxycycline, minocycline, and tigecycline, respectively.

SOC	PT	Doxycycline			Minocycline			Tigecycline		
		Risk signal	N	ROR (95%CI)	Risk signal	N	ROR (95%CI)	Risk signal	N	ROR (95%CI)
Gastrointestinal disorders	vomiting	●	**56**	**1.82 (1.39-2.37)**	●	38	0.75 (0.54-1.03)	●	**26**	**3.81 (2.57-5.64)**
	nausea	●	**32**	**1.83 (1.29-2.59)**	●	**48**	**1.69 (1.27-2.25)**	●	**28**	**7.33 (5.01-10.72)**
	colitis ulcerative	●	**30**	**9.17 (6.38-13.17)**	●	2	0.37 (0.09-1.47)	●	0	0.00
	dysphagia	●	**25**	**8.31 (5.59-12.35)**	●	5	1.01 (0.42-2.42)	●	2	2.88 (0.72-11.55)
	oesophageal ulcer	●	**23**	**389.61 (233.96-648.80)**	●	1	48.26 (6.11-381.02)	●	0	0.00
	oesophagitis	●	**19**	**55.53 (34.76-88.72)**	●	1	1.66 (0.23-11.85)	●	0	0.00
	abdominal pain upper	●	**19**	**2.33 (1.49-3.67)**	●	11	0.83 (0.46-1.50)	●	1	0.54 (0.08-3.82)
	pancreatitis	●	1	0.47 (0.07- 3.37)	●	1	1.47 (0.61-3.53)	●	**6**	**12.72 (5.68-28.48)**
	pancreatitis acute	●	3	2.14 (0.69- 6.66)	●	4	1.76 (0.66-4.71)	●	**7**	**22.39 (10.60-47.31)**
Nervous system disorders	headache	●	28	1.34 (0.92-1.94)	●	**78**	**2.33 (1.86-2.91)**	●	0	0.00
	idiopathic intracranial hypertension	●	12	14.62 (8.25-25.94)	●	**69**	**58.18 (45.17-74.92)**	●	0	0.00
	intracranial pressure increased	●	**17**	**13.58 (8.39-21.98)**	●	**28**	**13.96 (9.57-20.36)**	●	0	0.00
	dizziness	●	12	1.30 (0.74-2.30)	●	**28**	**1.88 (1.30-2.73)**	●	0	0.00
Psychiatric disorders	depression	●	**30**	**3.71 (2.58-5.32)**	●	11	0.83 (0.46-1.49)	●	0	0.00
	suicidal ideation	●	**22**	**3.15 (2.07-4.80)**	●	6	0.52 (0.23-1.17)	●	0	0.00
	suicide attempt	●	**16**	**2.28 (1.39-3.73)**	●	3	0.26 (0.08-0.81)	●	0	0.00
Hepatobiliary disorders	cholangitis sclerosing	●	**26**	**190.93 (123.62-294.89)**	●	2	7.18 (1.77-29.05)	●	0	0.00
	drug-induced liver injury	●	7	5.85 (2.78-12.33)	●	**48**	**26.23 (19.57-35.16)**	●	0	0.00
	autoimmune hepatitis	●	3	10.53 (3.37-32.95)	●	**35**	**90.72 (62.96-130.73)**	●	0	0.00
	hepatotoxicity	●	3	2.19 (0.71-6.81)	●	**8**	**3.62 (1.81-7.27)**	●	**21**	**71.55 (46.05-111.18)**
Skin and subcutaneous tissue disorders	drug reaction with eosinophilia and systemic symptoms	●	13	4.21 (2.44-7.28)	●	**136**	**29.64 (24.85-35.36)**	●	1	1.42 (0.20-10.07)
	neutrophilic dermatosis	●	**23**	**1818.21 (840.46-3933.45)**	●	0	0.00	●	0	0.00
	photosensitivity reaction	●	**16**	**26.09 (15.81-43.03)**	●	3	2.91 (0.93-9.06)	●	0	0.00
	urticaria	●	10	0.92 (0.50-1.72)	●	**40**	**2.30 (1.68-3.15)**	●	5	2.05 (0.85-4.94)
	dermatitis	●	4	2.28 (0.85- 610.00)	●	4	1.41 (0.53-3.75)	●	5	12.68 (5.25-30.64)
Endocrine disorders	hyperthyroidism	●	13	33.49 (19.17-58.50)	●	**58**	**111.12 (83.17-148.46)**	●	0	0.00
	thyroiditis	●	1	7.12 (0.99-51.08)	●	**50**	**439.78 (296.79-651.67)**	●	0	0.00
Blood and lymphatic system disorders	lymphadenopathy	●	1	0.48 (0.07-3.40)	●	**27**	**8.15 (5.56-11.94)**	●	0	0.00
	neutropenia	●	6	0.90 (0.40- 2.01)	●	2	0.18 (0.05-0.74)	●	**5**	**3.33 (1.38-8.04)**
	thrombocytopenia	●	2	0.39 (0.10- 1.55)	●	13	1.56 (0.90-2.69)	●	**9**	**7.82 (4.04-15.12)**
	bone marrow failure	●	10	5.19 (2.78- 9.68)	●	1	0.32 (0.04-2.25)	●	**8**	**18.47 (9.17-37.20)**
Musculoskeletal and connective tissue disorders	arthralgia	●	15	2.22 (1.34-3.70)	●	**63**	**5.87 (4.57-7.55)**	●	0	0.00
	lupus-like syndrome	●	3	12.52 (4.00-39.25)	●	**43**	**146.32 (103.47-206.93)**	●	0	0.00
	arthropathy	●	0	0.00	●	2	1.64 (0.41-6.56)	●	**6**	**35.74 (15.91-80.26)**
Respiratory, thoracic and mediastinal disorders	dyspnoea	●	**23**	**1.78 (1.18-2.69)**	●	20	0.95 (0.61-1.48)	●	4	1.36 (0.51-3.64)
General disorders and administration site conditions	pyrexia	●	18	0.60 (0.38-0.95)	●	**77**	**1.60 (1.28-2.01)**	●	5	0.73 (0.30-1.77)
	fatigue	●	14	1.10 (0.65-1.86)	●	**40**	**1.95 (1.43-2.66)**	●	6	2.08 (0.93-4.66)
	multiple organ dysfunction syndrome	●	4	1.10 (0.41-2.92)	●	**34**	**5.85 (4.16-8.21)**	●	4	4.85 (1.81-12.99)
Injury, poisoning and procedural complications	intentional overdose	●	**16**	**1.82 (1.12-2.98)**	●	1	0.07 (0.01-0.49)	●	0	0.00
Investigations	alanine aminotransferase increased	●	2	0.42 (0.11-1.70)	●	**33**	**4.39 (3.11-6.19)**	●	3	2.82 (0.91-8.77)
	aspartate aminotransferase increased	●	3	0.71 (0.23-2.20)	●	**31**	**4.59 (3.22-6.55)**	●	1	1.04 (0.15-7.42)
	weight decreased	●	11	1.73 (0.95-3.12)	●	**27**	**2.63 (1.80-3.84)**	●	2	1.38 (0.34-5.53)
	transaminases increased	●	7	3.57 (1.70-7.50)	●	18	5.71 (3.58-9.09)	●	**6**	**13.57 (6.06-30.40)**
	blood cholesterol increased	●	0	0.00	●	4	2.81 (1.05-7.52)	●	**8**	**41.05 (20.33-82.87)**
Metabolism and nutrition disorders	hypertriglyceridaemia	●	0	0.00	●	6	5.48 (2.45-12.27)	●	**17**	**117.12 (71.66-191.43)**
	hyperkalaemia	●	0	0.00	●	0	0.00	●	**5**	**22.77 (9.41-55.10)**
	decreased appetite	●	14	1.63 (0.96- 2.76)	●	17	1.22 (0.76-1.96)	●	**7**	**3.61 (1.71-7.61)**
Eye disorders	papilloedema	●	7	7.56 (3.59-15.94)	●	**40**	**28.25 (20.48-38.95)**	●	0	0.00
Immune system disorders	jarisch-herxheimer reaction	●	**29**	**795.54 (467.85-1352.73)**	●	0	0.00	●	0	0.00

● : PT with a significant risk signal; ● : PT without a significant risk signal.

To facilitate comparison, we listed the ROR for the corresponding PTs of each drug, with bold text indicating that the PT is among the top 20 for the drug.

(PT: preferred term; AE: adverse event; SOC: system organ class; ROR: reporting odds ratio; CI: confidence intervals).

#### 3.2.1 Gastrointestinal disorders

Gastrointestinal disorders in this study commonly presented with vomiting, nausea, ulcerative colitis, dysphagia, esophageal ulcer, esophagitis, upper abdominal pain, pancreatitis, and acute pancreatitis. Doxycycline was associated with all of these conditions except pancreatitis and acute pancreatitis, showing RORs of 1.82, 1.83, 9.17, 8.31, 389.61, 55.53, and 2.33, respectively, which were higher than those associated with minocycline. Minocycline only exhibited a risk for nausea, with an ROR of 1.69. Tigecycline was associated with vomiting, nausea, pancreatitis, and acute pancreatitis, with RORs of 3.81, 7.33, 12.72, and 22.39, respectively.

#### 3.2.2 Nervous system disorders

Nervous system disorders in this study primarily manifested as headache, idiopathic intracranial hypertension (IIH), increased intracranial pressure (ICP), and dizziness. Minocycline was associated with all of these risks, exhibiting RORs of 2.33, 58.18, 13.96, and 1.88, respectively, which were higher than those associated with doxycycline. Doxycycline was specifically linked to IIH and ICP, with RORs of 14.62 and 13.58, respectively. No significant signals were observed for tigecycline.

**FIGURE 3:**
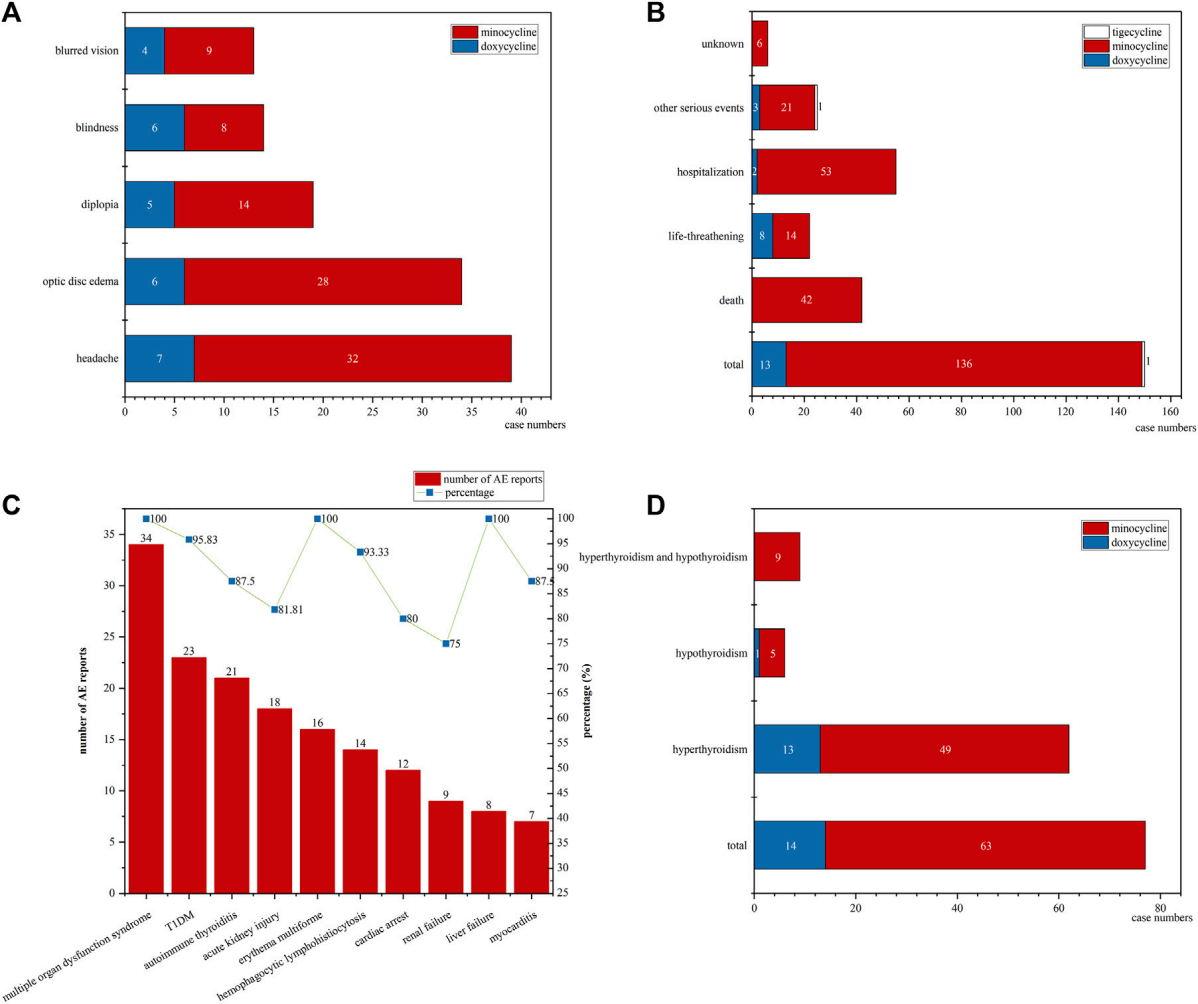
The details of IIH/ICP, DRESS, and thyroid dysfunction cases: **(A)** The co-occurring AEs in IIH/ICP cases; **(B)** The outcomes distribution in all DRESS cases; **(C)** The number of co-occurring AEs and their respective percentages of the total reports for each AE in minocycline-associated DRESS cases; **(D)** The distribution of hyperthyroidism and hypothyroidism among thyroid dysfunction cases. (IIH: idiopathic intracranial hypertension; ICP: increased intracranial pressure; DRESS: drug reactions with eosinophilia and systemic symptoms; AE: adverse event).

In pediatric patients diagnosed with either IIH or ICP, a total of 117 cases were identified, as detailed in Table 3 and illustrated in Figure 3A. Among these, 28 were linked to doxycycline, while 89 were associated with minocycline. Females, accounting for 78.63% of the cases, were affected more frequently. The median age of these patients was 15 years. The median time to onset of the symptoms was 31 days for minocycline and 53 days for doxycycline. The co-occurring AEs in these patients included headache in 39 patients (doxycycline: 7, minocycline: 32), optic disc edema in 34 patients (doxycycline: 6, minocycline: 28), diplopia in 19 patients (doxycycline: 5, minocycline: 14), blindness in 14 patients (doxycycline: 6, minocycline: 8), and blurred vision in 13 patients (doxycycline: 4, minocycline: 9).

**TABLE 3:** The characteristics of pediatric patients treated with doxycycline, minocycline, and tigecycline, including the outcomes of IIH/ICP, suicidal ideation/suicide attempts, DRESS, thyroid dysfunction, and dental staining (specific to children under 8 years old).

Characteristics	IIH/ICP	Suicidal ideation/suicide attempts	DRESS	Thyroid dysfunction	Dental staining under 8 years old
Number					
total	117	44	150	77	7
doxycycline	28	35	13	14	7
minocycline	89	8	136	63	0
doxycycline + minocycline	0	1	0	0	0
tigecycline	0	0	1	0	0
Gender					
Female	92 (78.63%)	22 (50.00%)	86 (57.33%)	47 (61.04%)	5 (71.43%)
Male	24 (20.51%)	22 (50.00%)	64 (42.67%)	30 (38.96%)	2 (28.57%)
Missing	1 (0.85%)	0	0	0	0
Median age, (y)	15 [13,16]	15 [14,16]	15 [14,17]	16 [16,16]	4 [3.5,5.5]
Median time to the onset, (d)					
total	53 [17,172]				
doxycycline	151 [72,196]				
minocycline	31 [16,122]	9 [7.5,12]	36 [20,49]		

(IIH: idiopathic intracranial hypertension; ICP: increased intracranial pressure; DRESS: drug reactions with eosinophilia and systemic symptoms).

#### 3.2.3 Psychiatric disorders

In our study, psychiatric disorders were predominantly characterized by depression, suicidal ideation, and suicide attempt. These risks were exclusively associated with doxycycline, with RORs of 3.71, 3.15, and 2.28, respectively. No significant risk signals were identified for minocycline and tigecycline.

For pediatric patients experiencing suicidal ideation and suicide attempts, the characteristics are detailed in Table 3. In total, 44 cases were reported, with 35 cases linked to doxycycline, 8 cases to minocycline, and one case associated with both medications. Fortunately, there were no fatalities among these children. The male-to-female ratio was 1:1. The median age of these patients was 15 years, and the youngest was 11 years old. Notably, the median onset time of symptoms associated with minocycline was only 9 days (95% CI: 7.5–12 days). Unfortunately, the data related to the onset time for doxycycline were incomplete.

#### 3.2.4 Hepatobiliary disorders

Hepatobiliary disorders in this study typically presented as sclerosing cholangitis, drug-induced liver injury, autoimmune hepatitis, and hepatotoxicity. For minocycline, the primary risks identified included drug-induced liver injury, autoimmune hepatitis, and hepatitis, with RORs of 26.23, 90.72, and 3.62, respectively. These RORs were notably higher compared to those associated with doxycycline. The identified risks for doxycycline included sclerosing cholangitis, drug-induced liver injury, and autoimmune hepatitis, with RORs of 190.93, 5.85, and 10.53, respectively. Tigecycline was specifically associated with a risk of hepatotoxicity, with an ROR of 71.55.

#### 3.2.5 Skin and subcutaneous tissue disorders

In our study, skin and subcutaneous tissue disorders were typically characterized by drug reactions with eosinophilia and systemic symptoms (DRESS), neutrophilic dermatosis, photosensitivity reaction, urticaria, and dermatitis. Among the evaluated drugs, tigecycline was only associated with a risk of dermatitis. Minocycline exhibited stronger AE signals compared to doxycycline in DRESS and urticaria risk signals, with RORs of 29.64 *versus* 4.21 for DRESS and 2.30 *versus* 0.92 for urticaria. Additionally, unlike minocycline and tigecycline, doxycycline also showed risk signals for neutrophilic dermatosis and photosensitivity reaction, with RORs of 1818.21 and 26.09, respectively.

Focusing on pediatric DRESS cases, we detail the results in Table 3 and illustrate them in Figures 3B,C. Our research identified a total of 150 cases: 13 associated with doxycycline, 136 with minocycline, and only 1 with tigecycline, accounting for 0.38%, 13.86%, and 0.71% of the total AE reports for each drug, respectively. Females represented the majority of affected patients, comprising 57.33% of the cases. The median age was 15 years. For minocycline cases, the median time to onset of DRESS symptoms was 36 days (95% CI: 20–49 days). The outcomes of these cases are shown in Figure 3B. Doxycycline was not associated with any fatalities but was associated with 8 life-threatening cases. In stark contrast, minocycline had a more severe impact, being linked to 42 deaths, which accounted for 30.88% of the drug’s DRESS cases, and 14 life-threatening cases.

In the minocycline-related DRESS cases, our study also analyzed their co-occurring AEs, with the results displayed in Figure 3C. We observed 34 instances of multiple organ dysfunction syndrome, 23 of Type 1 Diabetes Mellitus (T1DM), 21 of autoimmune thyroiditis, 18 of acute kidney injury, 16 of erythema multiforme, 14 of hemophagocytic lymphohistiocytosis, 12 of cardiac arrest, 9 of renal failure, 8 of liver failure, and 7 of myocarditis. Remarkably, these instances corresponded to 100%, 95.83%, 87.50%, 81.81%, 100%, 93.33%, 80.00%, 75.00%, 100.00%, and 87.50% of the total reports for each PT. Interestingly, although we observed 38 cases of autoimmune hepatitis and 46 of lupus-like syndrome, none of these conditions were noted in the minocycline-related DRESS cases.

#### 3.2.6 Endocrine disorders

In our study, the most prominent risk signals in endocrine disorders were hyperthyroidism and thyroiditis. The RORs for these conditions in association with minocycline were 111.12 and 439.78, respectively, which were significantly higher than those for doxycycline. For doxycycline, the primary risk signal was hyperthyroidism, with an ROR of 33.49. No significant risk signals were observed for tigecycline.

For thyroid dysfunction, including both hyperthyroidism and hypothyroidism, the details are presented in Table 3 and illustrated in Figure 3D. A total of 77 cases were identified. Of these, 14 were related to doxycycline and 63 to minocycline. The doxycycline-related cases comprised 13 cases of hyperthyroidism and 1 of hypothyroidism. The minocycline-related cases included 49 with hyperthyroidism, 5 with hypothyroidism, and 9 with both conditions. Females, accounting for 61.04%, were more frequently affected. In addition, the median age was 16 years.

### 3.3 Dental staining risks in children under 8 years old linked to tetracyclines

Focused on children under 8 years old, our study assessed tetracycline-related dental staining risks. In this age group, we collected 103 doxycycline-associated cases, 15 minocycline-associated cases, and 71 tigecycline-associated cases in total. The characteristics of dental staining within this age group are detailed in Table 3. Specifically, we identified 7 instances linked to doxycycline (ROR = 107.16%, 95% CI: 49.67–231.18), with the median age being 4 years (95% CI: 3.5–5.5 years); no cases were reported for minocycline and tigecycline. However, due to incomplete data and inherent limitations of the FAERS database, the duration of these treatments and the permanence of the dental discoloration remain unclear.

## 4 Discussion

In this study, we employed the ROR method to detect adverse reaction signals from the FAERS database, aiming to assess the safety of tetracyclines in pediatric populations.

From January 2005 to September 2023, we observed a predominantly gradual increase in the number of AE reports per 3-year period, underscoring the critical need for ongoing monitoring and evaluation of the safety of these drugs in children. Additionally, our analysis revealed that both doxycycline and minocycline were reported more frequently in female children, consistent with previous research (Martins et al., 2021). Notably, the severity of adverse reactions attributed to minocycline significantly exceeded those to doxycycline, aligning with prior studies (Smith and Leyden, 2005; Tasina et al., 2011; Lebrun-Vignes et al., 2012; Martins et al., 2021). Furthermore, we discovered that the fatalities associated with tigecycline accounted for 23.57% of the cases in our study.

Previous studies in adult populations have indicated that tigecycline is associated with an increased risk of all-cause mortality (McGovern et al., 2013; Shen et al., 2015; Pfizer Inc, 2020). In pediatric care, tigecycline is predominantly utilized for treating infections caused by MDR/XDR pathogens, with most of these patients being in the Intensive Care Unit (ICU). The severity of MDR/XDR infections and the underlying diseases may lead to higher mortality rates in immunocompromised patients, particularly in pediatric patients (Song et al., 2018). Previous reports indicate that the all-cause mortality rate in children treated with tigecycline varies between 18% and 40% (Ozkaya-Parlakay et al., 2020; Song et al., 2018; Zeng et al., 2017). In our study, given that 81.81% of tigecycline-related fatal cases involved PTs such as drug ineffective, drug ineffective for unapproved indication, treatment failure, drug resistance, intentional product use issue, multiple drug resistance, and product use issue, we are unable to assess the risk of death in children using tigecycline, and further research is needed. Additionally, the limited number of reports on tigecycline also restricted us to fully evaluate its safety in pediatric cases.

After the disproportionality analysis, it was observed that, apart from the risks of psychiatric disorder, the other 23 SOCs were all documented in their respective FDA prescribing information (Patheon Pharmaceuticals Inc, 2017; Mayne Pharma, 2022; Pfizer Inc, 2020). It is important to note that while the FDA prescribing information does not mention psychiatric risks associated with doxycycline, the New Zealand Medicines and Medical Devices Safety Authority (Medsafe) has documented depression, anxiety, and hallucination as rare side effects of doxycycline (Medsafe, 2023). The prevalent adverse reactions align with the findings from prior research: gastrointestinal responses are mainly associated with doxycycline and tigecycline, whereas minocycline is more frequently linked to neurological disorders and diseases of the skin and subcutaneous tissues.

### 4.1 Psychiatric risks in children

Our research has indicated a potential increase in psychiatric risks associated with the use of doxycycline in children, which is not currently mentioned in its FDA prescribing information. Specifically, our findings suggested a possible link between doxycycline and symptoms such as depression, suicidal ideation, and suicide attempt. To our knowledge, our study appears to be the first to identify the psychiatric risks of tetracyclines in pediatric patients. Previous case reports documented several severe psychiatric reactions to doxycycline in adults. Dyer described a 19-year-old female student from Cambridge University who developed paranoia and tragically committed suicide after taking doxycycline to prevent malaria (Dyer, 2020). Additionally, Healy et al. detailed three cases: two males, neither with a history of mental illness or substance abuse, committed suicide after taking doxycycline for 6 days and 8 weeks, respectively. Genetic testing revealed the former had the CYP2C19*2 heterozygous genotype, which is associated with diminished cytochrome P450 enzyme activity. Intriguingly, his two brothers also suffered severe anxiety after using doxycycline, and the symptom resolved after discontinuation of the medication. The third case involved a 33-year-old woman who developed suicidal thoughts on the third day after taking doxycycline for recurrent acne, which subsided after discontinuing the medication (Atigari et al., 2013). It is important to note that these previous case reports predominantly involve doxycycline, with no reported cases yet involving minocycline.

Notably, while our findings indicated a correlation between doxycycline and increased psychiatric risks in children, establishing a direct cause remains challenging. This is partly due to confounding factors, such as existing psychiatric illnesses, the use of additional medications, the sensitive stage of adolescence, and the illnesses being treated with tetracyclines, which might have psychiatric implications. Previous studies have shown that conditions like acne and Lyme disease, which are often treated with these antibiotics, also increase psychiatric risks (Eichenfield et al., 2021; Fallon et al., 2021). Therefore, more thorough and methodical research is essential to fully understand the relationship between tetracyclines and psychiatric risks.

### 4.2 Risk of DRESS in children

Our study has shown that both doxycycline and minocycline may elevate the risk of DRESS, with minocycline demonstrating stronger AE signals compared to doxycycline. DRESS syndrome, a severe drug-induced hypersensitivity reaction, is typically characterized by rash, fever, blood test abnormalities, and internal organ damage. This condition affects children disproportionately more than adults, typically manifesting as a measles-like rash 2–8 weeks after the initiation of drug therapy. The syndrome carries a mortality risk of up to 10%, primarily due to complications such as liver damage or myocarditis (Manieri et al., 2023; Wei et al., 2023). Following the cessation of the causative medication, approximately 5.9%–16% of patients may suffer long-term effects, including organ dysfunction and autoimmune conditions like autoimmune thyroiditis, hepatitis, and T1DM (Wei et al., 2023). These ongoing symptoms might result from the formation of doxycycline/minocycline-melanin complexes. A study found that minocycline can still be detected in the blood 17 months after discontinuing minocycline treatment (Maubec et al., 2008).

Utilizing data from the French Pharmacovigilance Database and marketing authorization holders for the years 1985–2007, focused on the general population, previous research found that the prevalence of minocycline-induced DRESS varied between 4% and 8% (Lebrun-Vignes et al., 2012). Furthermore, earlier studies have shown that the mortality rate for DRESS is 5.4% in pediatric patients, compared to 10% in adults (Manieri et al., 2023; Wei et al., 2023). Our analysis revealed that 13.86% of pediatric cases associated with minocycline developed DRESS, with 30.88% of these cases resulting in fatalities in the FAERS database. However, the precise prevalence and mortality rate of minocycline-induced DRESS in children still necessitates further research, owing to the FAERS database’s inherent limitations and the scarcity of previous studies.

In our analysis of co-occurring AEs in minocycline-associated DRESS cases, we observed a significantly high number of multiple organ dysfunction syndrome, T1DM, and autoimmune thyroiditis, which are all long-term sequelae. However, studies detailing these long-term sequelae of minocycline-associated DRESS remain limited. Lan et al. documented a case of a 13-year-old girl who developed DRESS after minocycline treatment for acne, which led to a liver transplant and the diagnosis of autoimmune T1DM, requiring ongoing insulin therapy (Lan et al., 2016). Similarly, Brown et al. reported a 15-year-old female adolescent who developed DRESS 4 weeks after starting minocycline therapy for acne, resulting in autoimmune hyperthyroidism 7 weeks later and autoimmune T1DM 7 months after discontinuing minocycline therapy (Brown et al., 2009). Furthermore, our study found no instances of DRESS coinciding with autoimmune hepatitis or lupus-like syndrome, suggesting that minocycline could induce autoimmunity even without causing DRESS, aligning with previous findings (El-Hallak et al., 2008).

### 4.3 Thyroid dysfunction in children

In prior studies, numerous reports have highlighted thyroid pigment deposition associated with minocycline use, yet instances of thyroid dysfunction were seldom reported in these cases (Goto et al., 2022). Additionally, contrary to the common association of thyroid dysfunction with autoimmune thyroiditis, non-autoimmune thyroid dysfunctions have been less frequently reported (Benjamin and Calikoglu, 2007). Our research identified 77 cases of thyroid dysfunction, with 14 related to doxycycline and 63 to minocycline. Notably, none of these cases were reported to have autoimmune thyroiditis. Among the limited case reports available, instances involving adults were rare (Tacon et al., 2008), whereas adolescents were comparatively more frequent (Benjamin and Calikoglu, 2007; Pollock et al., 2016; Millington et al., 2019). Researchers Pollock et al. and Millington et al. reported three and nine adolescent cases, respectively, who developed thyroid dysfunction following acne treatment, with one case related to doxycycline and eleven to minocycline. Most patients’ thyroid function normalized within 4.5 months after stopping the medication. However, one case of minocycline-associated hypothyroidism persisted for more than 4.5 years after discontinuation (Pollock et al., 2016; Millington et al., 2019). Despite this, thyroid dysfunction warnings are not included in the package insert for doxycycline and are mentioned only as “cases of abnormal thyroid function have been reported” for minocycline (Patheon Pharmaceuticals Inc, 2017; Mayne Pharma, 2022).

### 4.4 IIH risk in children

IIH is a serious medical condition characterized by elevated intracranial pressure, predominantly affecting young, overweight women, presenting symptoms like headaches, pulsatile tinnitus, and visual issues including peripheral vision loss, transient vision blurring, and diplopia (Grech et al., 2020). In cases of IIH triggered by tetracyclines, both the duration of symptoms and outcomes vary considerably after medication discontinuation (Martins et al., 2021; Passi et al., 2022). While some reports indicate symptoms may resolve spontaneously after stopping the medication, there are also cases of permanent vision loss and persistent visual impairment (Somech et al., 1999; Paramo and Leishangthem, 2023). In our research, though the long-term outcome of these symptoms was beyond our study’s scope, we observed 14 cases of blindness linked to doxycycline (6 cases) and minocycline (8 cases), highlighting the potential severity of IIH’s impact.

### 4.5 Potential risks of dental discoloration from tetracyclines in children below 8 years

In our research on children below 8 years, we observed dental discoloration exclusively in cases treated with doxycycline (ROR = 107.16%, 95% CI: 49.67–231.18). However, both the duration of the treatment and the permanence of the staining remained uncertain. Contrary to our findings, a recent review involving 338 children under 8 years old found no significant difference in dental discoloration between the doxycycline-treated group and the control group (Stultz and Eiland, 2019). A case report also described reversible tooth staining in a 6-year-old girl treated with doxycycline (Joshi et al., 2020). In 2018, the American Academy of Pediatrics (AAP) revised their guidelines in the Red Book, stating that doxycycline can be administered for short durations (i.e., 21 days or less) regardless of the patient’s age. Additionally, the Red Book removed the warning about potential tooth discoloration for doxycycline, although it remains for tetracycline (American Academy of Pediatrics, 2018). Currently, doxycycline is recommended as the treatment of choice for Lyme disease and Rocky Mountain spotted fever in children of all ages (American Academy of Pediatrics, 2018; Meissner and Steere, 2022). With the increasing use of doxycycline in this age group, additional safety studies would be beneficial. As for minocycline and tigecycline, although our study did not detect a risk signal of tooth staining, there are related case reports in children under 8 years. Reported dental discoloration in a 7-year-old girl after treatment with minocycline as a root canal medication. Despite the improvement in the cervical shade following three walking bleaching procedures, the tooth did not return to its original shade (Kim et al., 2010). Zhu et al. observed mild yellow discoloration in two children under 8 years old after discontinuing tigecycline for 4 years (Zhu et al., 2021).

Our study has several limitations. Firstly, the FDA does not require a direct causal link between AEs and medications, limiting our ability to establish a definitive cause-and-effect relationship. Secondly, FAERS is a voluntary reporting system, resulting in potential gaps in data quality, accuracy, or completeness. Thirdly, the FDA does not receive a product’s all AE reports. The reports in the database may also be influenced by factors like product longevity and public awareness. Therefore, FAERS data cannot be used to calculate the incidence of an AE in the population. Fourthly, the ROR method we employed, although straightforward, is particularly sensitive to individual data points, and the statistic fluctuates greatly if the cell frequency is small. Hence, we focused on AEs with higher case counts to reduce false positives. Finally, our analysis was exclusively restricted to the safety signals of doxycycline, minocycline, and tigecycline in pediatric use, and other tetracyclines were excluded due to the limited number of reports.

## 5 Conclusion

Using real-world data from the FAERS, this study evaluated the safety profiles of three tetracyclines in pediatric patients. Our findings indicated that for pediatric patients, the majority of results were in line with the prescribing information and previous studies, and minocycline tended to cause more frequent and severe AEs than doxycycline. However, it is noteworthy that exceptions were found for psychiatric disorders and thyroid dysfunction associated with doxycycline, which are not mentioned in its prescribing information. Additionally, further safety studies on tigecycline are still needed for children. When prescribing tetracyclines to pediatric patients, a careful risk-benefit assessment is crucial. We advise closely monitoring mental health, immune function, thyroid, liver, eye, and dental health.

## Data Availability

The original contributions presented in the study are included in the article/Supplementary Material, further inquiries can be directed to the corresponding authors.
