# Supplementary material for: Safety profiles of doxycycline, minocycline, and tigecycline in pediatric patients: a real-world pharmacovigilance analysis based on the FAERS database
Source: Front Pharmacol. 2024 Jul 26;15:1413944. doi: 10.3389/fphar.2024.1413944 (PMC11317777; doi:10.3389/fphar.2024.1413944)
Supplement: Supplementary file 2 [file Table1.DOCX]

**Table S1.** Thorough drug name archive of tetracyclines

| **Drug classifications** | **Generic names** | **Brand names** | **Patent numbers** | **ATC codes** |
| --- | --- | --- | --- | --- |
| **Doxycyclines** | Doxycycline-Hyclate | VIBRA-TABS; Vibramycin; Vibramicina | [EP-00091409](https://edu-pharma.bcpmdata.com/database/1/detail/48/TtNfa4sBmjBxIaXiIRxI) | A01AB22; [J01AA02](https://db.yaozh.com/atc?atc_num=J01AA02) |
|  | Doxycycline | Oracea; Periostat; Apprilon | [WO-09808480](https://edu-pharma.bcpmdata.com/database/1/detail/48/U91ja4sBmjBxIaXizLu7) |  |
|  | Doxycycline | Atridox; ATRIGEL | WO-09101126 |  |
|  | Doxycycline-Hyclate | Doryx; Doryx MPC | [WO-03086366](https://edu-pharma.bcpmdata.com/database/1/detail/48/Jt5ja4sBmjBxIaXi9h_x) |  |
|  | Doxycycline-Hyclate | Acticlate | [WO-09509608](https://edu-pharma.bcpmdata.com/database/1/detail/48/2NRfa4sBmjBxIaXihSpc) |  |
|  | Doxycycline | Periostat | [EP-00195906](https://edu-pharma.bcpmdata.com/database/1/detail/48/H-Fla4sBmjBxIaXiSAYj) |  |
|  | Doxycycline | Zenavod | WO-2009032326 |  |
|  | Doxycycline-Hyclate |  | EP-00537559 |  |
| **Minocyclines** | Minocycline | Minocin | [US-03978000](https://edu-pharma.bcpmdata.com/database/1/detail/48/0dNfa4sBmjBxIaXiad3o) | A01AB23; [J01AA08](https://db.yaozh.com/atc?atc_num=J01AA08) |
|  | Minocycline-Hydrochloride | Solodyn | [US-05908838](https://edu-pharma.bcpmdata.com/database/1/detail/48/K8hba4sBmjBxIaXiezXM) |  |
|  | Minocycline-Hydrochloride | Zilxi | WO-2008032212 |  |
|  | Minocycline-Hydrochloride | Amzeeq | WO-2009072007 |  |
|  | Minocycline | Arestin | [WO-03082139](https://edu-pharma.bcpmdata.com/database/1/detail/48/W-Fla4sBmjBxIaXiSAYj) |  |
|  | Minocycline-Hydrochloride | Minocin | [WO-2011143503](https://edu-pharma.bcpmdata.com/database/1/detail/48/Qdpia4sBmjBxIaXiRF0e) |  |
| **Tigecyclines** | Tigecycline | Tygacil | EP-00536515 | J01AA12 |
